# Supplementary material for: Mesenchymal stem cell therapy induces FLT3L and CD1c+ dendritic cells in systemic lupus erythematosus patients
Source: Nat Commun. 2019 Jun 7;10:2498. doi: 10.1038/s41467-019-10491-8 (PMC6555800; doi:10.1038/s41467-019-10491-8)
Supplement: Supplementary file 1 — Supplementary [file 41467_2019_10491_MOESM1_ESM.pdf]

Mesenchymal stem cell therapy induces FLT3L and CD1c^{+} dendritic cells in systemic lupus erythematosus patients

Yuan et al.

S1 Gated on Lin$^-$HLA-DR$^+$CD11c$^+$DCs

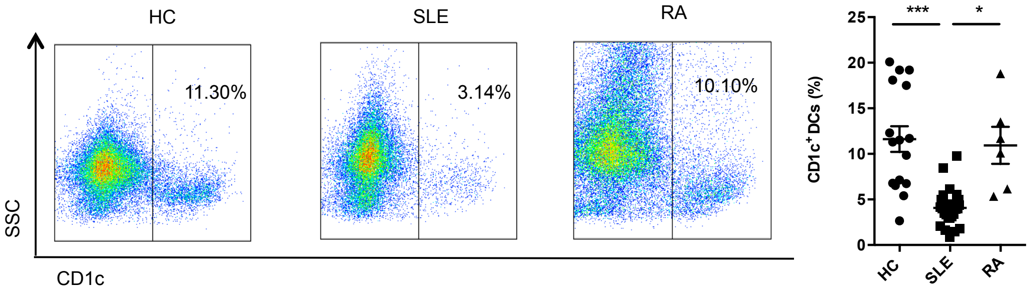

Supplementary Figure 1. The number of Lin$^-$HLA-DR$^+$CD11c$^+$CD1c$^+$DCs in PBMCs was compared among SLE patients (n=25), healthy controls (HC) (n=16) and rheumatic arthritis (RA) patients (n=6) by flow cytometry. The experiment was repeated ≥3 times (* $p < 0.05$, *** $p < 0.001$ by one-way ANOVA), SEM.

S2

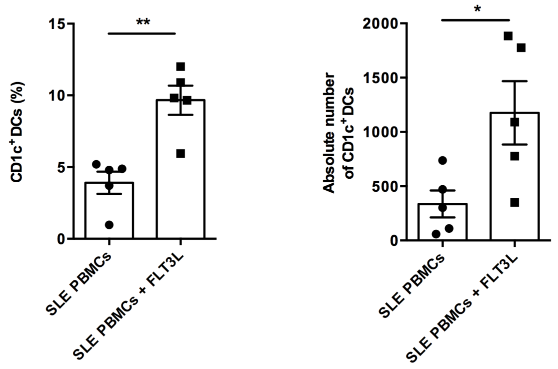

Supplementary Figure 2. PBMCs from SLE patients were stimulated with human recombinant FLT3L with 10ng/ml for 48h, the frequency and absolute number of Lin$^-$HLA-DR$^+$CD11c$^+$CD1c$^+$DCs were evaluated by flow cytometry (n=5). The experiment was repeated ≥3 times (* $p < 0.05$, ** $p < 0.01$ by t-test), SEM.

S3

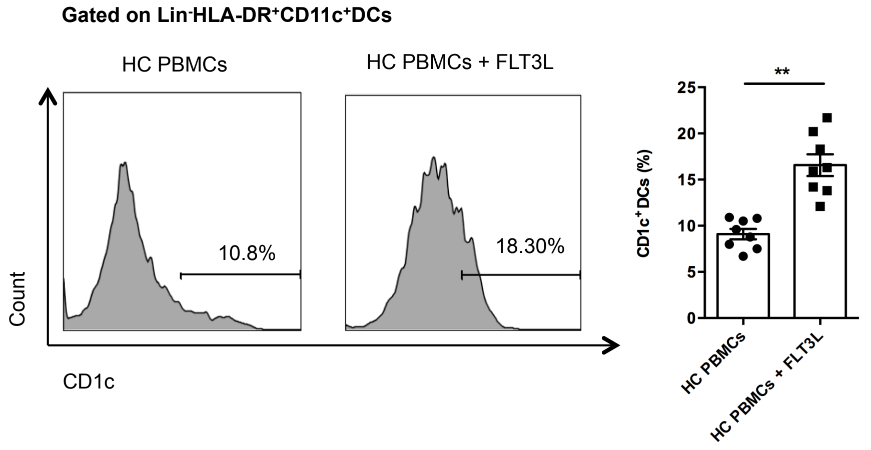

Supplementary Figure 3. PBMCs from healthy subjects were stimulated with human recombinant FLT3L with 10ng/ml for 48h, the frequency of Lin$^-$HLA-DR$^+$CD11c$^+$CD1c$^+$DCs was evaluated by flow cytometry (n=8). The experiment was repeated ≥3 times (** $p < 0.01$ by t-test), SEM.

S4

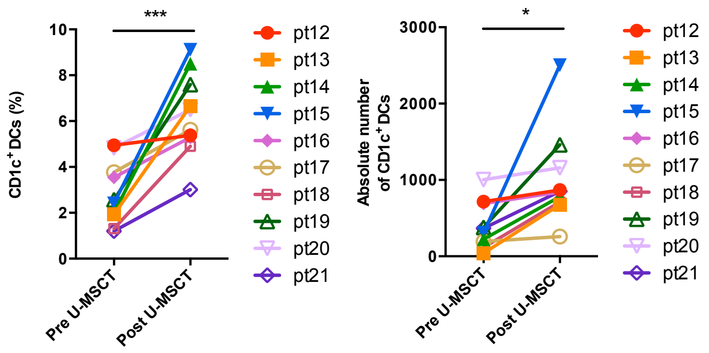

Supplementary Figure 4. Ten refractory lupus patients were given U-MSCT. Quantification of Lin$^-$HLA-DR$^+$CD11c$^+$CD1c$^+$DCs by flow cytometry before and 24h after U-MSCT (n=10). The experiment was repeated ≥3 times (* $p < 0.05$, *** $p < 0.001$ by t-test).

S5

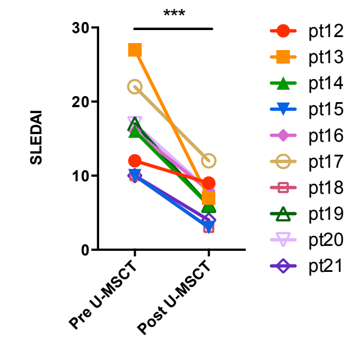

Supplementary Figure 5. Ten refractory lupus patients were given U-MSCT. The SLEDAI score was evaluated before and 1 month after U-MSCT (*n*=10). The experiment was repeated ≥3 times (*** $p < 0.001$ by *t*-test).

S6

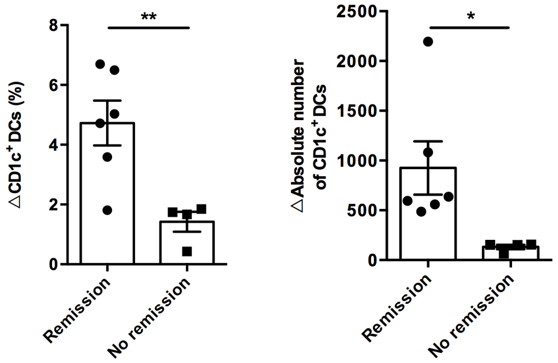

Supplementary Figure 6. Among 10 SLE patients receiving U-MSCT, 6 patients showed partial remission with SLEDAI scores < 8 at 1-month follow-up (remission group), and the other 4 patients showed no remission with SLEDAI scores ≥ 8 at their 1-month follow-up (no remission group). The increases of both frequency and absolute number of $CD1c^{+}$DCs after U-MSCT were compared between the two groups. △=post U-MSCT - pre U-MSCT. The experiment was repeated ≥3 times (* $p < 0.05$, ** $p < 0.01$ by *t*-test), SEM.

S7

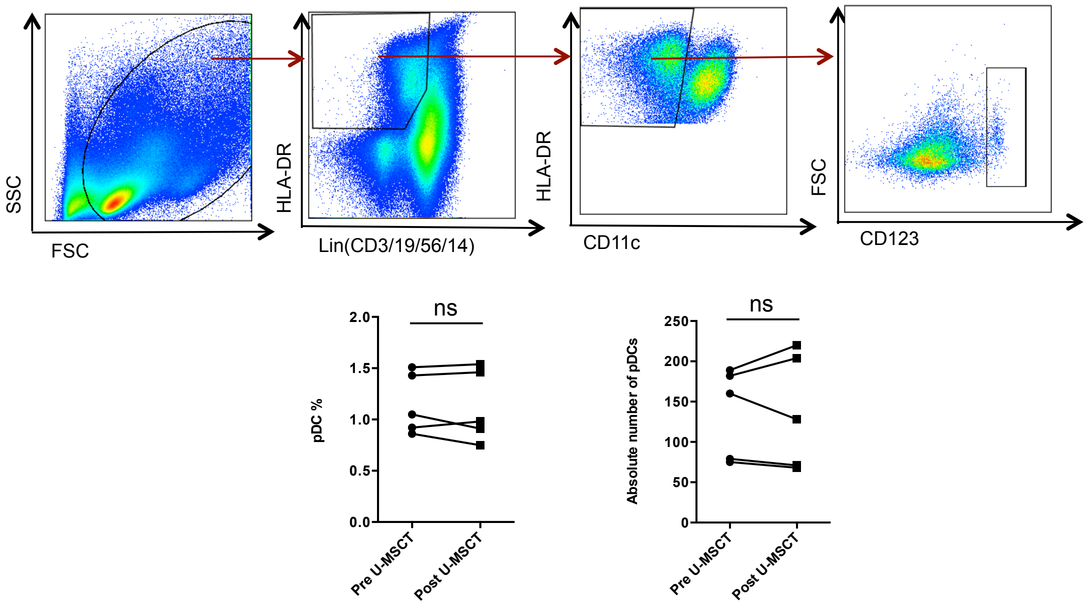

Supplementary Figure 7. FACS gating strategy used to identify pDCs, which were defined as Lin(CD3/19/56/14)$^-$HLA-DR$^+$CD11c$^-$CD123$^+$. The percentage and absolute number of pDCs were analyzed before and 24h after U-MSCT (*n*=5). The experiment was repeated ≥3 times (ns, not significant by *t*-test).

S8

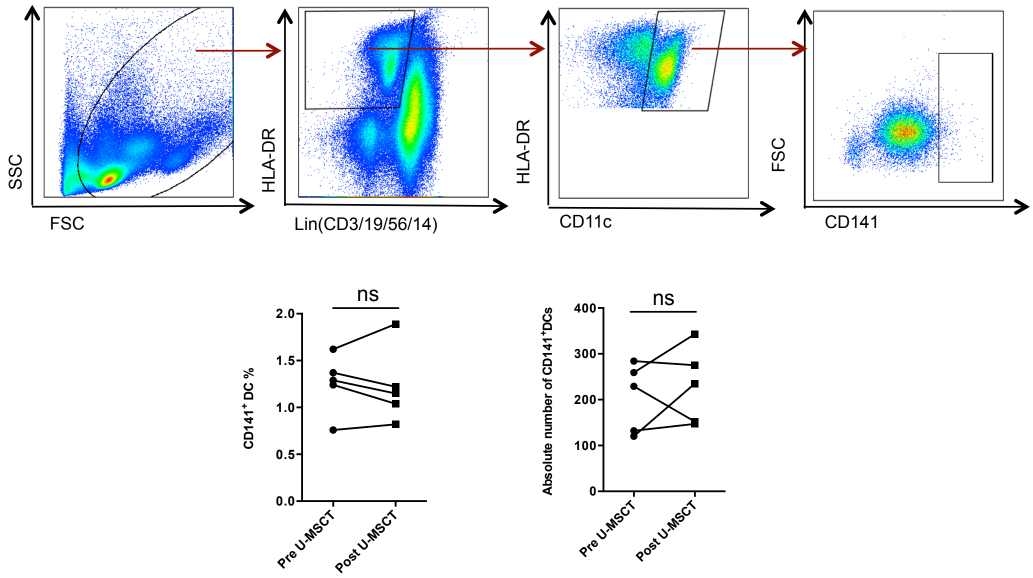

Supplementary Figure 8. FACS gating strategy used to identify CD141$^+$DCs, which were defined as Lin(CD3/19/56/14)$^-$HLA-DR$^+$CD11c$^+$CD141$^+$. The percentage and absolute number of CD141$^+$DCs were analyzed before and 24h after U-MSCT (*n*=5). The experiment was repeated ≥3 times (ns, not significant by *t*-test).

S9

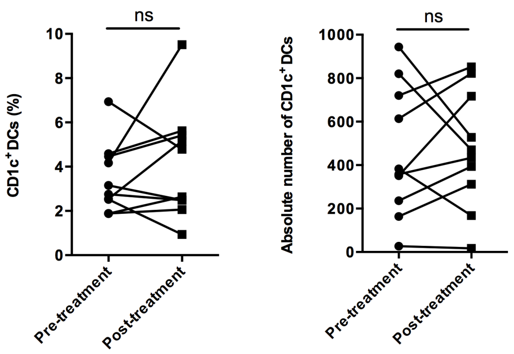

Supplementary Figure 9. The lupus nephritis patients who achieved partial remission after regular medication therapy were enrolled. The percentage and absolute number of Lin$^-$HLA-DR$^+$CD11c$^+$CD1c$^+$DCs in PBMCs were compared before and after treatment by flow cytometry (n=10). The experiment was repeated ≥3 times (ns, not significant by t-test), SEM.

S10

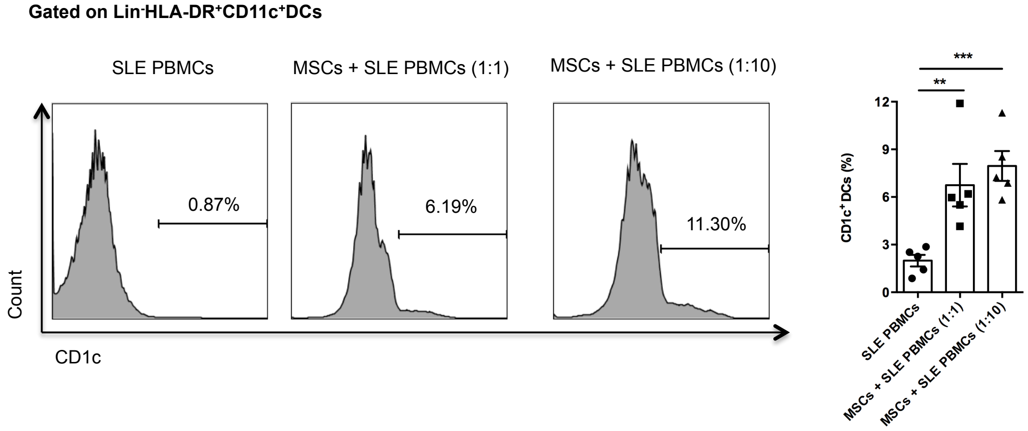

Supplementary Figure 10. The PBMCs from active lupus patients were co-cultured with UC-MSCs in different ratios (1:1 or 10:1) for 72h. The percentages of Lin$^-$HLA-DR$^+$CD11c$^+$CD1c$^+$DCs in PBMCs were evaluated by flow cytometry (n=5). The experiment was repeated ≥3 times (** $p < 0.01$, *** $p < 0.001$ by one-way ANOVA), SEM.

S11

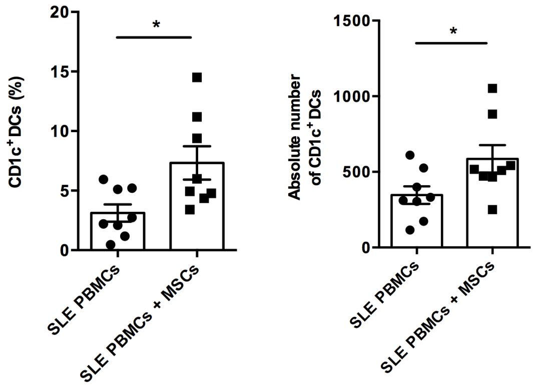

Supplementary Figure 11. The percentage and the absolute number of CD1c$^+$DCs were evaluated by flow cytometry when lupus PBMCs co-cultured with UC-MSCs for 72h (n=8). The experiment was repeated ≥3 times (* $p < 0.05$ by t-test), SEM.

S12

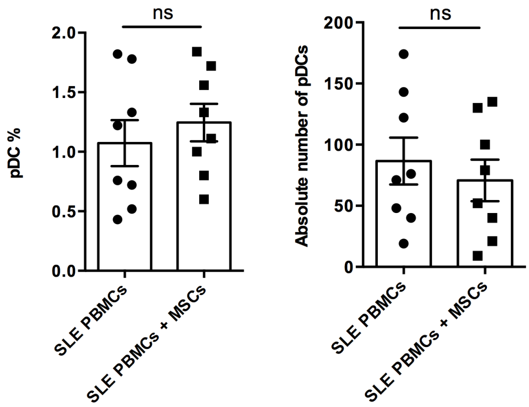

Supplementary Figure 12. The SLE PBMCs were co-cultured with UC-MSCs for 72h. The percentage and the absolute number of Lin$^-$HLA-DR$^+$CD11c$^-$ CD123$^+$ pDCs were evaluated by flow cytometry (n=8). The experiment was repeated ≥3 times (ns, not significant by t-test), SEM.

S13

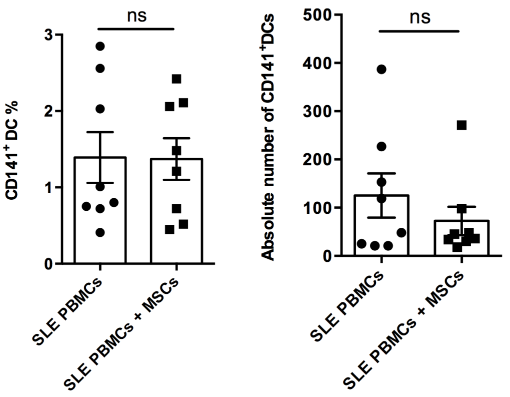

Supplementary Figure 13. The SLE PBMCs were co-cultured with UC-MSCs for 72h. The percentage and the absolute number of $Lin^{-}HLA\text{-}DR^{+}CD11c^{+}$ $CD141^{+}$DCs were evaluated by flow cytometry (*n*=8). The experiment was repeated ≥3 times (ns, not significant by *t*-test), SEM.

S14

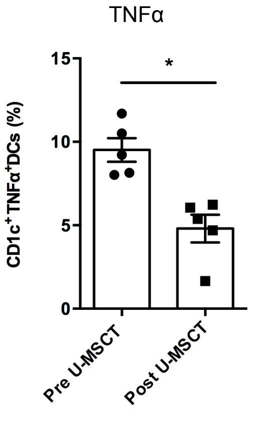

Supplementary Figure 14. Upon CD40L stimulation, the level of TNFα produced by $Lin^{-}HLA\text{-}DR^{+}CD11c^{+}CD1c^{+}$DCs was evaluated using flow cytometry before and 24h after U-MSCT (*n*=5). The experiment was repeated ≥3 times (* $p < 0.05$ by *t*-test), SEM.

S15

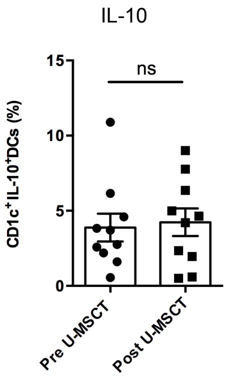

Supplementary Figure 15. Upon CD40L stimulation, the level of IL-10 produced by Lin$^-$HLA-DR$^+$CD11c$^+$CD1c$^+$DCs was evaluated using flow cytometry before and 24h after U-MSCT (n=10). The experiment was repeated ≥3 times (ns, not significant by t-test), SEM.

S16

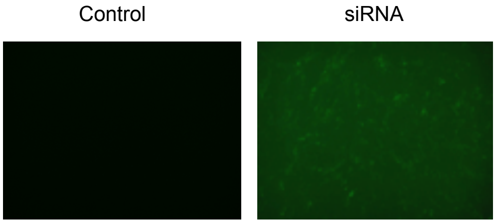

Supplementary Figure 16. FLT3L siRNA was transfected into UC-MSCs. The experiment was repeated ≥3 times.

S17

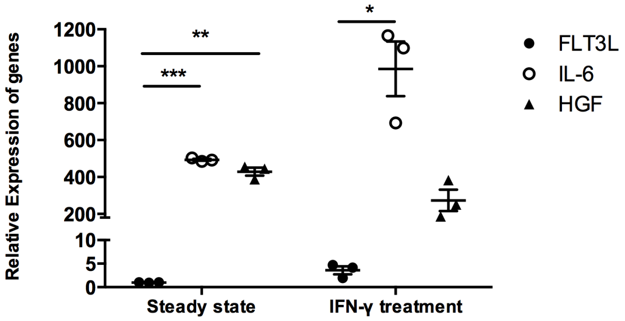

Supplementary Figure 17. The expressions of FLT3L, IL-6 and HGF in UC-MSCs were evaluated by real-time PCR in steady state and under IFN-γ treatment (n=3). The experiment was repeated ≥3 times (* $p < 0.05$, ** $p < 0.01$, *** $p < 0.001$ by one-way ANOVA), SEM.

S18

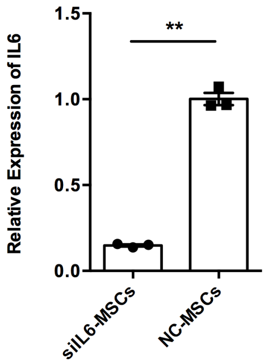

Supplementary Figure 18. The inhibition efficiency of IL-6 siRNA was assessed in UC-MSCs by real-time PCR (*n*=3). The experiment was repeated ≥3 times (** $p < 0.01$ by *t*-test), SEM. siIL6-MSCs = IL-6 siRNA treated MSCs, NC-MSCs = negative control siRNA treated MSCs.

S19

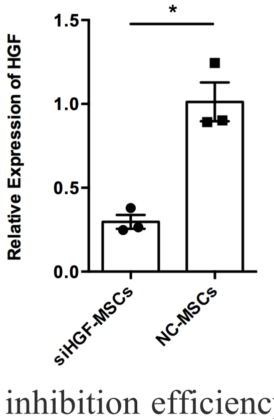

Supplementary Figure 19. The inhibition efficiency of HGF siRNA was assessed in UC-MSCs by real-time PCR (*n*=3). The experiment was repeated ≥3 times (* $p < 0.05$ by *t*-test), SEM. siHGF-MSCs = HGF siRNA treated MSCs, NC-MSCs = negative control siRNA treated MSCs.

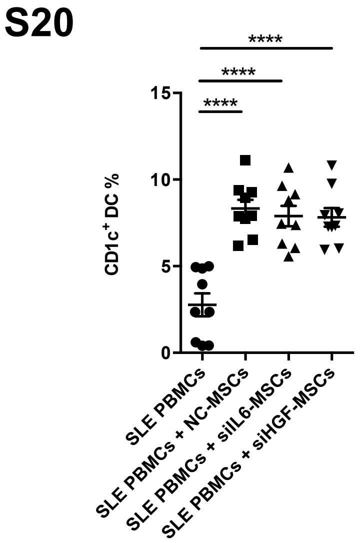

Supplementary Figure 20. SLE PBMCs were co-cultured with UC-MSCs, which were transfected with negative control siRNA or siRNA against IL-6/HGF. The percentage of $Lin^{-}HLA\text{-}DR^{+}CD11c^{+}CD1c^{+}$DCs in PBMCs was evaluated using flow cytometry (n=9). The experiment was repeated ≥3 times (**** $p < 0.0001$ by one-way ANOVA), SEM. siIL6-MSCs = IL-6 siRNA treated MSCs, siHGF-MSCs = HGF siRNA treated MSCs, NC-MSCs = negative control siRNA treated MSCs.

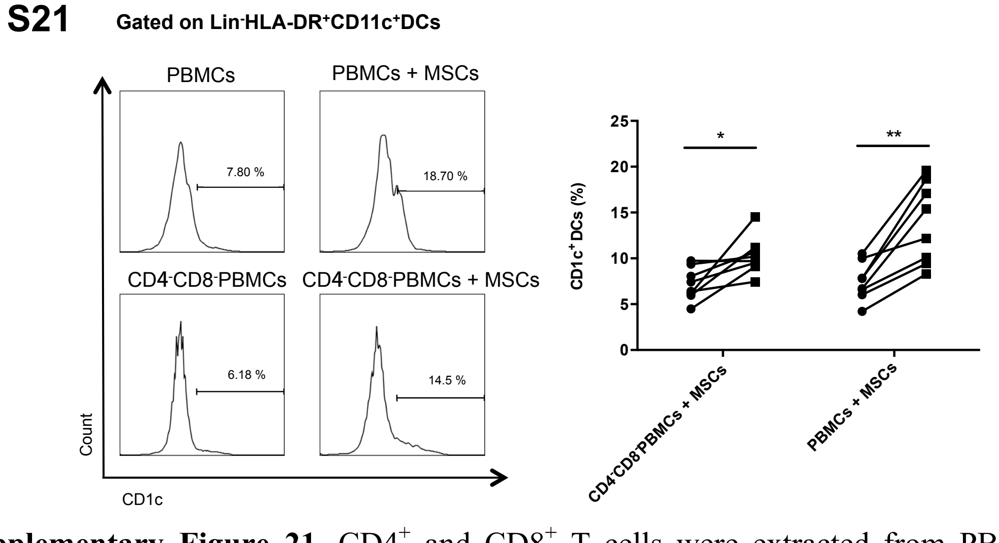

Supplementary Figure 21. $CD4^{+}$ and $CD8^{+}$ T cells were extracted from PBMCs respectively using micro-beads. PBMCs or T cell-depleted PBMCs were then co-cultured with UC-MSCs for 72h. The percentage of $Lin^{-}HLA\text{-}DR^{+}CD11c^{+}CD1c^{+}$DCs in PBMCs was evaluated using flow cytometry (n=8). The experiment was repeated ≥3 times (* $p < 0.05$, ** $p < 0.01$ by t-test).

S22

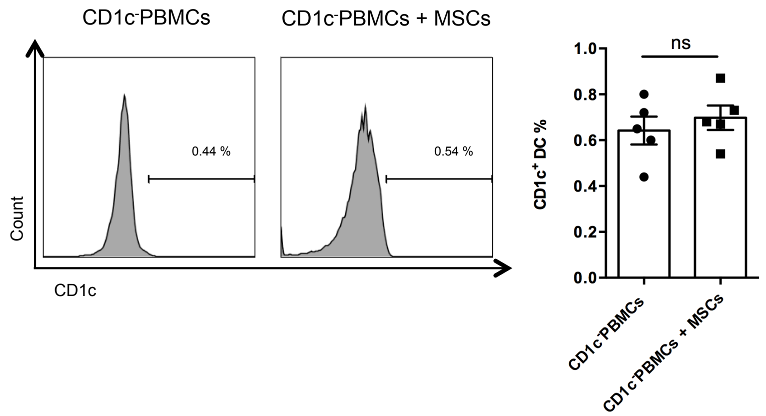

Supplementary Figure 22. CD1c^{+}DCs were extracted from PBMCs using micro-beads. Then the CD1c^{-}PBMCs were co-cultured with UC-MSCs for 72h. The frequency of Lin^{-}HLA-DR^{+}CD11c^{+}CD1c^{+}DCs in PBMCs was evaluated using flow cytometry (*n*=5). The experiment was repeated ≥3 times (ns, not significant by *t*-test), SEM.

S23

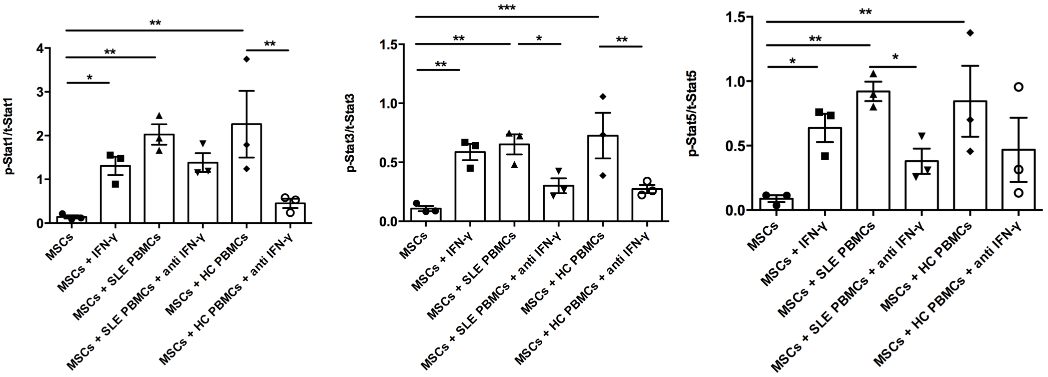

Supplementary Figure 23. Quantification of western blot band density using ImageJ for the Figure 5f (*n*=3). The uncropped scans of western blots are presented in Source Data file. The experiment was repeated ≥3 times (* $p < 0.05$, ** $p < 0.01$, *** $p < 0.001$ by one-way ANOVA), SEM.

S24

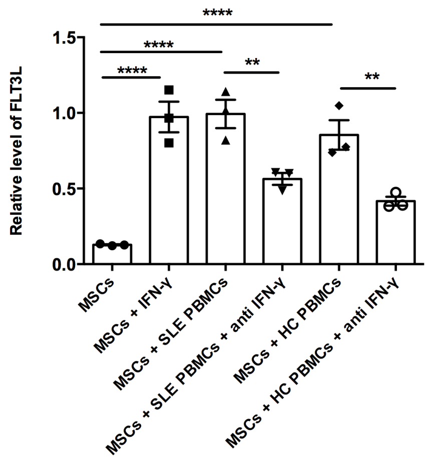

Supplementary Figure 24. Quantification of western blot band density using ImageJ for the Figure 5g (n=3). The uncropped scans of western blots are presented in Source Data file. The experiment was repeated ≥3 times (** $p < 0.01$, **** $p < 0.0001$ by one-way ANOVA), SEM.

Supplementary Table 1. Patients' baseline clinical characteristics and medications before/after U-MSCT

Patient	Age/sex	Disease duration (months)	Baseline SLEDAI	Clinical manifestation	Treatment before U-MSCT	Treatment 1-mo after U-MSCT
12	32/F	48	14	LN, A, H, ANA+, anti-dsDNA+, anti-SM+	Pred 20mg/d, CYC 0.8gm/mo, MMF 1.5gm/d, Tacrolimus 2mg/d, HCQ 0.2gm/d (12 mo)	Pred 7.5mg/d, MMF 1.5gm/d, HCQ 0.2gm/d
13	28/F	120	27	LN, NP-SLE, A, H, F, C, anti-dsDNA+	Pred 40mg/d, Tacrolimus 3mg/d (6 mo)	Pred 15mg/d, Tacrolimus 3mg/d
14	23/F	48	16	LN, A, H, F, C, ANA+, anti-dsDNA+, anti-SM+	Pred 20mg/d, Tacrolimus 2mg/d, HCQ 0.2gm/d (8 mo)	Pred 10mg/d, Tacrolimus 2mg/d, HCQ 0.2gm/d
15	22/F	48	10	LN, H, ANA+, anti-SM+	Pred 40mg/d (1 mo), then tapered to 20 mg/d, CYC 0.8gm/mo, MMF 1.5gm/d, HCQ 0.2gm/d (12 mo)	Pred 10mg/d, MMF 0.75gm/d
16	43/F	264	16	LN, A, C, H	Pred 30mg/d, CYC 0.8gm/mo, HCQ 0.2gm/d (8 mo)	Pred 5mg/d, HCQ 0.2gm/d
17	40/F	96	22	LN, NP-SLE, A, dysopia, H ANA+, anti-SM+,	Pred 20mg/d, CYC 0.8gm/mo, HCQ 0.2gm/d (14 mo)	Pred 10mg/d, HCQ 0.2gm/d
18	25/F	108	10	LN, H, ANA+, anti-dsDNA+, anti-SM+	Pred 30mg/d, MMF 1.5gm/d, HCQ 0.2gm/d (7 mo)	Pred 15mg/d, MMF 0.5gm/d, HCQ 0.2gm/d
19	50/F	60	17	LN, A, C, H, ANA+, anti-dsDNA+, anti-SM+	Pred 30mg/d, MMF 1.5gm/d, LEF 20mg/d, HCQ 0.2gm/d (14 mo)	Pred 10mg/d, LEF 20mg/w
20	16/F	60	17	LN, A, C, H, ANA+, anti-dsDNA+	Pred 60mg/d (1 mo), then tapered to 20mg/d, MMF 1.5gm/d, Tacrolimus 2mg/d (12 mo)	Pred 5mg/d, MMF 1.5gm/d
21	30/F	96	10	LN, H, ANA+, anti-dsDNA+, anti-SM+	Pred 15mg/d, MMF 1.5gm/d, Tacrolimus 2mg/d, HCQ 0.2gm/d (8 mo)	Pred 5mg/d, MMF 1.5gm/d, HCQ 0.2gm/d

A, arthralgia; ANA, antinuclear antibody; anti-dsDNA, anti double strand DNA antibody; C, cytopenia; CYC, cyclophosphamide; d, day; F, febrile; H, hypocomplementemia; HCQ, hydroxychloroquine; LEF, leflunomide; LN, lupus nephritis; MMF, mycophenolate mofetil; mo, month; NP-SLE, neuropsychiatric lupus; P, polyserositis; Pred, prednisone; U-MSCT, UC-MSCs transplantation; w, week.

Supplementary Table 2. Baseline clinical characteristics and medications for the other SLE patients who donated blood for the cell culture and flow cytometry experiments

Patient	Age/sex	Disease duration (months)	Clinical manifestation	Medication
1	25/F	36	C, H, ANA+, anti-dsDNA+, anti-SM+	Pred, HCQ
2	23/F	24	LN, A, C, F, ANA+, anti-dsDNA+, anti-SM+	Pred, MMF, CYC, HCQ
3	63/F	120	LN, A, H, ANA+, anti-dsDNA+, anti-SM+	Pred, CYC, HCQ
4	34/M	228	F, C, ANA+, anti-dsDNA+, anti-SM+	Pred, HCQ
5	16/M	121	F, C, ANA+, anti-dsDNA+, anti-SM+	Pred, HCQ
6	45/M	96	C, H, P, ANA+	Pred, HCQ
7	43/F	96	C, F, H, P, ANA+, anti-dsDNA+	Pred, HCQ
8	25/F	180	LN, A, C, F, ANA+	Pred, CYC, HCQ
9	45/F	1	LN, H, ANA+, anti-dsDNA+, anti-SM+	Pred
10	29/F	48	A, C, H, ANA+, anti-dsDNA+, anti-SM+	Pred, HCQ
11	44/M	12	LN, H, P, ANA+, anti-dsDNA+, anti-SM+	Pred, CYC, HCQ
12	23/F	2	LN, A, ANA+, anti-dsDNA+, anti-SM+	Pred, cyclosporin, HCQ
13	42/F	4	C, H, ANA+, anti-dsDNA+, anti-SM+,	Pred
14	49/F	180	LN, A, ANA+, anti-dsDNA+, anti-SM+	Pred, CYC
15	38/F	132	LN, C, ANA+, anti-dsDNA+, anti-SM+	Pred, LEF, HCQ
16	45/F	108	A, C, H, ANA+, anti-dsDNA+	Pred, AZA
17	41/F	240	A, C, H, P, ANA+	Pred, HCQ
18	53/M	12	A, F, H, ANA+	Pred, HCQ
19	25/F	61	A, C, H, ANA+, anti-dsDNA+, anti-SM+	Pred, cyclosporin, HCQ
20	48/F	6	LN, A, F, C, H, ANA+, anti-SM+	Pred, MMF, CYC, HCQ
21	20/F	30	LN, A, F, H, P, ANA+, anti-dsDNA+, anti-SM+	Pred, CYC, MMF, HCQ
22	52/M	21	LN, A, F, H, P, ANA+, anti-dsDNA+, anti-SM+	Pred, CYC, Tacrolimus, HCQ
23	56/F	120	A, C, ANA+, anti-dsDNA+, anti-SM+	Pred
24	36/F	12	LN, C, H, ANA+, anti-dsDNA+, anti-SM+	Pred, LEF
25	35/F	132	H, P, V, ANA+, anti-dsDNA+, anti-SM+	Pred, Tacrolimus, HCQ
26	21/F	47	A, P, ANA+, anti-dsDNA+, anti-SM+	Pred, HCQ
27	28/F	39	A, C, F, ANA+, anti-dsDNA+, anti-SM+	Pred, HCQ
28	27/F	144	LN, C, H, ANA+	Pred, AZA
29	26/F	42	LN, F, H, ANA+, anti-dsDNA+, anti-SM+	Pred, CYC, HCQ
30	37/F	240	A, H, ANA+, anti-dsDNA+, anti-SM+	Pred, HCQ
31	24/F	12	F, H, ANA+, anti-dsDNA+, anti-SM+	Pred
32	13/M	1	LN, C, F, H, ANA+, anti-SM+	Pred, LEF, HCQ
33	33/F	120	LN, C, H, ANA+, anti-dsDNA+, anti-SM+	Pred, CYC, HCQ
34	23/F	2	LN, F, C, H, ANA+, anti-dsDNA+, anti-SM+	Pred, CYC, AZA
35	45/F	120	LN, C, F, H, ANA+	Pred, cyclosporin, HCQ
36	16/F	96	LN, C, H, ANA+, anti-dsDNA+, anti-SM+	Pred, LEF, HCQ
37	25/M	2	LN, C, H, ANA+, anti-SM+	Pred, MMF, HCQ

38	26/F	120	A, H, ANA+, anti-SM+	Pred, HCQ
39	30/M	84	LN, ANA+, anti-SM+	Pred, LEF, HCQ
40	38/F	36	A, C, ANA+, anti-dsDNA+	Pred
41	13/M	10	LN, H, ANA+, anti-dsDNA+	Pred, MMF
42	22/F	36	LN, A, H, ANA+, anti-dsDNA+, anti-SM+	Pred, MMF, LEF, HCQ
43	20/F	1	H, ANA+, anti-dsDNA+, anti-SM+	Pred
44	41/F	4	LN, C, H, ANA+, anti-dsDNA+, anti-SM+	Pred, LEF, HCQ
45	34/M	228	LN, ANA+, anti-dsDNA+, anti-SM+	Pred, MMF, CYC
46	22/F	6	H, P, ANA+, anti-SM+	Pred, HCQ
47	26/F	72	H, ANA+, anti-dsDNA+, anti-SM+	Pred, HCQ
48	38/F	13	LN, C, H, ANA+, anti-dsDNA+, anti-SM+	Pred, CYC, HCQ
49	14/F	1	A, C, H, ANA+, anti-SM+	Pred, HCQ
50	15/M	72	A, F, H, ANA+, anti-dsDNA+, anti-SM+	Pred, HCQ
51	28/F	24	H, ANA+, anti-dsDNA+, anti-SM+	Pred
52	26/F	72	LN, C, F, H, ANA+, anti-dsDNA+, anti-SM+	Pred, Tacrolimus
53	45/F	96	A, ANA+, anti-SM+	Pred, LEF, HCQ
54	36/F	108	C, F, H, ANA+, anti-dsDNA+, anti-SM+	Pred, LEF, HCQ
55	49/F	36	C, ANA+, anti-dsDNA+, anti-SM+	Pred, HCQ
56	49/F	264	LN, F, C, H	Pred, MMF, HCQ
57	31/F	120	H, ANA+, anti-dsDNA+, anti-SM+	Pred, HCQ
58	35/F	96	LN, H, ANA+, anti-dsDNA+, anti-SM+	Pred, CYC, HCQ
59	34/F	96	C, H, ANA+, anti-dsDNA+	Pred, HCQ
60	28/M	60	F, ANA+, anti-dsDNA+	Pred, HCQ
61	30/M	1	LN, C, H, ANA+, anti-dsDNA+, anti-SM+	Pred, CYC, HCQ
62	16/F	26	LN, H, ANA+, anti-dsDNA+, anti-SM+	Pred, Tacrolimus, HCQ
63	28/F	60	H, ANA+, anti-dsDNA+	Pred
64	30/F	120	LN, A, F, H, PAH, ANA+	Pred, MMF, HCQ
65	38/F	36	LN, A, C, H, ANA+, anti-dsDNA+	Pred, MMF, CYC
66	21/F	60	LN, H, ANA+, anti-dsDNA+, anti-SM+	Pred, Tacrolimus, HCQ
67	39/F	60	F, H, ANA+, anti-SM+	Pred, HCQ
68	33/F	12	LN, A, ANA+, anti-dsDNA+, anti-SM+	Pred, Tacrolimus
69	35/F	120	LN, H, ANA+, anti-dsDNA+, anti-SM+	Pred, Tacrolimus
70	38/F	0.5	F, H, ANA+, anti-dsDNA+, anti-SM+	Pred, HCQ
71	22/F	84	A, H, ANA+, anti-dsDNA+, anti-SM+	Pred, LEF, HCQ
72	37/F	240	A, F, ANA+	Pred, HCQ
73	44/F	2	C, F, H, ANA+, anti-dsDNA+, anti-SM+	Pred
74	27/F	72	H, ANA+	Pred, HCQ
75	38/F	153	LN, H, P,	Pred, MMF
76	37/F	72	C, H, ANA+	Pred
77	40/F	84	A, C, F	Pred, HCQ
78	24/F	35	LN, F, C, H, ANA+, anti-dsDNA+	Pred, MMF, HCQ
79	55/F	120	A, F, ANA+	Pred, HCQ
80	34/F	96	LN, A, C, H, ANA+, anti-SM+	Pred, MMF, HCQ

81	33/F	84	LN, A, F, P	Pred, LEF, HCQ
82	43/F	312	A, P, ANA+	Pred, HCQ
83	26/M	39	LN, H, ANA+, anti-dsDNA+, anti-SM+	Pred, MMF, HCQ
84	30/F	72	LN, A, ANA+, anti-dsDNA+	Pred, Tacrolimus, HCQ
85	27/F	48	P, ANA+, anti-dsDNA+, anti-SM+	Pred, HCQ
86	33/F	108	A, C, F, ANA+	Pred
87	32/F	180	LN, H, P	Pred, CYC
88	25/F	96	LN, C, H, ANA+, anti-dsDNA+	Pred, CYC, HCQ
89	21/F	60	A, F, ANA+, anti-dsDNA+, anti-SM+	Pred, HCQ
90	28/F	120	LN, C, H, ANA+, anti-SM+	Pred, MMF, LEF, HCQ
91	19/F	24	LN, F, H, P, ANA+, anti-dsDNA+	Pred, CYC, HCQ
92	37/F	168	LN, A, H, ANA+, anti-dsDNA+	Pred, MMF, HCQ
93	25/F	5	A, H, ANA+, anti-SM+	Pred, LEF, HCQ
94	36/M	4	C, F, H, anti-SM+	Pred, LEF, HCQ
95	30/F	120	LN, P, ANA+, anti-dsDNA+, anti-SM+	Pred, MMF, HCQ
96	37/F	72	LN, C, H, P, ANA+, anti-dsDNA+	Pred, CYC
97	26/F	72	LN, H, ANA+	Pred, MMF, HCQ
98	32/F	1	C, H, ANA+, anti-dsDNA+, anti-SM+	No medication
99	41/M	120	LN, F, H, ANA+, anti-dsDNA+, anti-SM+	Pred, MMF, CYC
100	25/M	108	C, H, ANA+, anti-dsDNA+, anti-SM+	Pred, HCQ
101	46/F	102	F, H, ANA+, anti-dsDNA+	Pred, HCQ
102	46/F	2	F, H, ANA+, anti-dsDNA+, anti-SM+	Pred, HCQ
103	41/F	96	A, H	Pred, LEF, HCQ
104	47/F	60	LN, A, C, F, H, P, ANA+, anti-dsDNA+	Pred, CYC, MMF, HCQ
105	35/F	72	LN, A, H, ANA+, anti-dsDNA+, anti-SM+	Pred, CYC, LEF, HCQ
106	36/F	108	A, H, ANA+, anti-dsDNA+, anti-SM+	Pred, LEF
107	49/F	60	LN, C, F, H, NP-SLE, ANA+, anti-dsDNA+, anti-SM+	Pred, MMF, cyclosporin
108	56/F	84	C, H, ANA+	Pred, CYC
109	38/F	24	LN, A, C, H, anti-dsDNA+	Pred, MMF, Tacrolimus
110	32/F	156	F, H, ANA+, anti-dsDNA+, anti-SM+	Pred, HCQ
111	50/F	192	LN, A, anti-dsDNA+, anti-SM+	Pred, MMF, HCQ
112	57/F	240	LN, H, ANA+, anti-dsDNA+, anti-SM+	Pred, CYC, HCQ
113	47/F	2	A, C, F, P, ANA+	Pred, cyclosporin, HCQ
114	51/F	120	H, ANA+, anti-dsDNA+, anti-SM+	Pred, CYC, HCQ
115	60/F	108	LN, C, ANA+	Pred, CYC, CTX, HCQ
116	47/F	72	LN, A, F, H, ANA+, anti-dsDNA+, anti-SM+	Pred, CYC, HCQ
117	31/F	4	C, F, H, P, ANA+	Pred, Tacrolimus, LEF, HCQ
118	33/F	132	LN, F, H, P, ANA+, anti-dsDNA+, anti-SM+	Pred, MMF, Tacrolimus, HCQ
119	32/F	96	H, P, ANA+, anti-dsDNA+, anti-SM+	Pred, CYC, LEF, HCQ
120	33/F	156	A, C, H, ANA+, anti-dsDNA+, anti-SM+	Pred, CYC, cyclosporin, HCQ
121	28/F	132	LN, F, H, ANA+, anti-SM+	Pred, HCQ
122	52/F	4	LN, H, ANA+, anti-SM+	Pred, Tacrolimus, HCQ
123	59/F	84	LN, A, C, F, H, NP-SLE, ANA+, anti-dsDNA+,	Pred, MMF, Tacrolimus, HCQ

			anti-SM+	
124	18/F	0.5	C, F, H, ANA+, anti-dsDNA+, anti-SM+	Pred, LEF, HCQ
125	62/F	264	C, H, ANA+, anti-SM+	Pred, HCQ
126	62/F	168	LN, A, C, F, H, ANA+, anti-dsDNA+	Pred, CYC, HCQ
127	50/F	10	Severe thrombocytopenia, C, H, ANA+	Pred, cyclosporin, HCQ
128	41/F	96	Severe thrombocytopenia, A, C, P, ANA+, anti-SM+	Pred, CYC, HCQ
129	73/F	408	F, H, P, ANA+	Pred
130	34/F	96	LN, F, H	Pred, HCQ
131	60/F	120	LN, P, ANA+, anti-dsDNA+, anti-SM+	Pred, CYC, LEF, HCQ
132	29/F	48	LN, C, H, P, ANA+, anti-dsDNA+, anti-SM+	Pred, CYC, HCQ
133	18/M	1	LN, C, F, H, P, V, ANA+, anti-dsDNA+, anti-SM+	Pred, CYC, MMF, LEF
134	36/F	12	LN, A, C, F, H, P, ANA+, anti-dsDNA+	Pred, MMF, LEF
135	36/F	24	LN, C, H, ANA+, anti-dsDNA+	Pred, CYC, LEF
136	30/F	0.5	LN, F, H, P, ANA+, anti-dsDNA+	Pred, CYC, HCQ
137	34/F	2	LN, C, P, ANA+	Pred, CYC, LEF, HCQ
138	61/F	120	LN, A, H, P, ANA+, anti-SM+	Pred, MMF, HCQ
139	16/F	1	LN, A, C, F, H, ANA+, anti-dsDNA+, anti-SM+	Pred, MMF, HCQ
140	57/F	120	LN, A, C, H, P, ANA+	Pred, CYC, HCQ
141	44/M	8	LN, A, C, F, ANA+, anti-dsDNA+	Pred, MMF, LEF
142	63/F	48	LN, C, H, P, ANA+, anti-SM+	Pred, MMF, LEF
143	46/F	72	LN, A, C, H, NP-SLE, P, ANA+, anti-dsDNA+, anti-SM+	Pred, CYC, Tacrolimus, LEF, HCQ
144	49/F	228	LN, H, ANA+, anti-dsDNA+	Pred, CYC, MMF, HCQ
145	27/F	24	A, F, H, ANA+, anti-dsDNA+, anti-SM+	Pred, Tacrolimus, HCQ

A, arthralgia; Aza, azathioprine; ANA, antinuclear antibody; anti-dsDNA, anti double strand DNA antibody; C, cytopenia; CYC, cyclophosphamide; F, febrile; H, hypocomplementemia; HCQ, hydroxychloroquine; LEF, leflunomide; LN, lupus nephritis; MMF, mycophenolate mofetil; NP-SLE, neuropsychiatric lupus; P, polyserositis; PAH, pulmonary arterial hypertension；Pred, prednisone; U-MSCT, UC-MSCs transplantation; V, vasculitis.

Supplementary Table 3. Patients' ID in each figure

Figure	Patient ID
1cdefghi	1-25
2abcd	26-37
2e	38-45
3m	46-53
3nop	54-61,101-105
3uvwx	124-131
4f	62-73
4g	142-145
4h	115-123
5bc	74-80
5de	81-87
5fg	79,80,88
5h	89-95
S2	101-105
S9	132-141
S10	96-100
S11	124-131
S12	124-131
S13	124-131
S20	106-114
S23	79,80,88
S24	79,80,88
