# Supplementary material for: Mesenchymal stem cell therapy induces FLT3L and CD1c+ dendritic cells in systemic lupus erythematosus patients
Source: Nat Commun. 2019 Jun 7;10:2498. doi: 10.1038/s41467-019-10491-8 (PMC6555800; doi:10.1038/s41467-019-10491-8)
Supplement: Supplementary file 3 — Reporting Summary [file 41467_2019_10491_MOESM3_ESM.pdf]

Corresponding author(s): DBPR NCOMMS-18-26759C

Reporting Summary

Nature Research wishes to improve the reproducibility of the work that we publish. This form provides structure for consistency and transparency in reporting. For further information on Nature Research policies, see Authors & Referees and the Editorial Policy Checklist.

Statistical parameters

When statistical analyses are reported, confirm that the following items are present in the relevant location (e.g. figure legend, table legend, main text, or Methods section).

n/a	Confirmed	
☐	☒	The exact sample size (*n*) for each experimental group/condition, given as a discrete number and unit of measurement
☐	☒	An indication of whether measurements were taken from distinct samples or whether the same sample was measured repeatedly
☐	☒	The statistical test(s) used AND whether they are one- or two-sided<br>*Only common tests should be described solely by name; describe more complex techniques in the Methods section.*
☒	☐	A description of all covariates tested
☒	☐	A description of any assumptions or corrections, such as tests of normality and adjustment for multiple comparisons
☐	☒	A full description of the statistics including central tendency (e.g. means) or other basic estimates (e.g. regression coefficient) AND variation (e.g. standard deviation) or associated estimates of uncertainty (e.g. confidence intervals)
☐	☒	For null hypothesis testing, the test statistic (e.g. *F*, *t*, *r*) with confidence intervals, effect sizes, degrees of freedom and *P* value noted<br>*Give P values as exact values whenever suitable.*
☒	☐	For Bayesian analysis, information on the choice of priors and Markov chain Monte Carlo settings
☒	☐	For hierarchical and complex designs, identification of the appropriate level for tests and full reporting of outcomes
☒	☐	Estimates of effect sizes (e.g. Cohen's *d*, Pearson's *r*), indicating how they were calculated
☐	☒	Clearly defined error bars<br>*State explicitly what error bars represent (e.g. SD, SE, CI)*

Our web collection on statistics for biologists may be useful.

Software and code

Policy information about availability of computer code

Data collection	No software was used
Data analysis	SPSS16.0 software and GraphPad Prism 4.3 was used to analyze the data. FlowJo X 10.0.7r2 software was used for flow cytometry result analysis.

For manuscripts utilizing custom algorithms or software that are central to the research but not yet described in published literature, software must be made available to editors/reviewers upon request. We strongly encourage code deposition in a community repository (e.g. GitHub). See the Nature Research guidelines for submitting code & software for further information.

Data

Policy information about availability of data

All manuscripts must include a data availability statement. This statement should provide the following information, where applicable:

- Accession codes, unique identifiers, or web links for publicly available datasets
- A list of figures that have associated raw data
- A description of any restrictions on data availability

The authors declare that all data supporting the findings of this study are available in the article and its Supplementary Information files or are available from the corresponding author on request.

Field-specific reporting

Please select the best fit for your research. If you are not sure, read the appropriate sections before making your selection.

☒ Life sciences ☐ Behavioural & social sciences ☐ Ecological, evolutionary & environmental sciences

For a reference copy of the document with all sections, see nature.com/authors/policies/ReportingSummary-flat.pdf

Life sciences study design

All studies must disclose on these points even when the disclosure is negative.

Sample size	Based on previous and preliminary studies within our lab,we predicted the reported sample sizes (145 SLE patients and 78 healthy controls for in vitro experiment, 21 SLE patients for in vivo experiment) would be sufficient to ensure adequate power.
Data exclusions	No data exclusion.
Replication	All attempts at replication were successful.
Randomization	The SLE patients were consecutively enrolled according to the following inclusion criteria: All patients fulfilled the 1997 revised criteria of the American College of Rheumatology for SLE. Twenty-one SLE patients refractory to conventional therapies were enrolled in an U-MSCT trial, with an SLEDAI score of more than or equal to 8 or with at least one British Isles Lupus Assessment Group (BILAG) grade A or at least two BILAG grade B manifestations. Refractory to treatment was defined as lack of response to conventional immunosuppressive drugs (cyclophosphamide 500-750 mg/m2/month, mycophenolate mofetil ≥1000 mg/day, leflunomide 20 mg/day, azathioprine 100 mg/day, tacrolimus ≥ 2 mg/day, alone or in combination for more than 6 months) or continued daily doses of at least 20 mg of prednisone or its equivalent. The healthy controls were included with efforts to match age and gender. This is a case-control study, hence, randomization is not applicable, and it is is not relevant.
Blinding	As mentioned above, it is a case-control study, hence,blinding is not applicable, and it is is not relevant.

Reporting for specific materials, systems and methods

Materials & experimental systems

n/a	Involved in the study
☒	☐ Unique biological materials
☐	☒ Antibodies
☒	☐ Eukaryotic cell lines
☒	☐ Palaeontology
☒	☐ Animals and other organisms
☐	☒ Human research participants

Methods

n/a	Involved in the study
☒	☐ ChIP-seq
☐	☒ Flow cytometry
☒	☐ MRI-based neuroimaging

Antibodies

Antibodies used

The antibodies are described below.
FITC-human hematopoietic lineage cocktail, eBioscience
PE-anti-human CD1c, eBioscience
PE-anti-human CD135 (FLT3),eBioscience
PE-anti-human CD123, eBioscience
PE-anti-human CD141, BD Biosciences
APC-anti-human CD11c, eBioscience
PE-Cy7-anti-human HLA-DR, eBioscience
BV711- anti-human CD40, BioLegend
PE-Dazzle594-anti-human CD80, BioLegend
PE-Dazzle594-anti-human TNFα, BioLegend
APC-Cy7-anti-human CD83, BioLegend
APC-Cy7-anti-human CD1c, BioLegend
BV605-anti-human CD86, BioLegend
BV421-anti-human IL-10, BioLegend
PerCP-Cy5.5-anti-human CD11c, BioLegend
Cell Proliferation Dye eFluor™ 670 , eBioscience

FITC-Annexin V, eBioscience
7AAD,eBioscience
Anti-Flt3 ligand monoclonal antibody, Abcam
Anti-IFN-γ monoclonal antibody,R&D Systems
Isotype control (mouse IgG1κ, mouse IgG2a, mouse IgG2b, Rat IgG1κ) , eBioscience
Anti-p-stat1/stat1, Abcam
Anti-p-stat3/stat3, Abcam
Anti-p-stat5/stat5, Abcam
Anti-FLT3L, Abcam

Validation

All antibodies were validated by manufacturer.

Human research participants

Policy information about studies involving human research participants

Population characteristics

The average age of the 145 SLE patients included in the in vitro experiment was 35.8 years old， with 125 female and 20 male. One patient was newly-onset SLE without any medication, the other 144 patients underwent steroid therapy, and 96 underwent immunosuppressant therapy including cyclophosphamide, mycophenolate mofetil, cyclosporine, azathioprine, leflunomide, tacrolimus, etc.
The average age of the 21 SLE patients included in the in vivo experiment was 32.5 years old, with 18 female and 3 male. They were refractory to conventional therapies, and underwent UC-MSCs transplantation.
The average of the 78 healthy controls included in the in vitro experiment was 34.0 years old， with 66 female and 12 male.

Recruitment

The SLE patients were consecutively enrolled according to the following inclusion criteria: All patients fulfilled the 1997 revised criteria of the American College of Rheumatology for SLE. Twenty-one SLE patients refractory to conventional therapies were enrolled in an U-MSCT trial, with an SLEDAI score of more than or equal to 8 or with at least one British Isles Lupus Assessment Group (BILAG) grade A or at least two BILAG grade B manifestations. Refractory to treatment was defined as lack of response to conventional immunosuppressive drugs (cyclophosphamide 500-750 mg/m2/month, mycophenolate mofetil ≥1000 mg/day, leflunomide 20 mg/day, azathioprine 100 mg/day, tacrolimus ≥ 2 mg/day, alone or in combination for more than 6 months) or continued daily doses of at least 20 mg of prednisone or its equivalent. The healthy controls were included with efforts to match age and gender. Patients were excluded from the study if they had the following conditions: (1) uncontrolled infection, such as infection, including pneumonia (bacterial, virus, or fungal), pulmonary tuberculosis, hepatitis B and C, skin infection, central nervous system infection; (2) severe organ dysfunction such as heart failure New York Heart Association functional classification III or IV, hepatic failure, renal failure, or respiratory failure; (3) woman who was pregnant or lactating, or a woman or man who intended to initiate a pregnancy in the following 6 months. This is the standard criteria of inclusion and exclusion for SLE, and no bias exists.

Flow Cytometry

Plots

Confirm that:

☐ The axis labels state the marker and fluorochrome used (e.g. CD4-FITC).

☒ The axis scales are clearly visible. Include numbers along axes only for bottom left plot of group (a 'group' is an analysis of identical markers).

☐ All plots are contour plots with outliers or pseudocolor plots.

☒ A numerical value for number of cells or percentage (with statistics) is provided.

Methodology

Sample preparation

Peripheral blood mononuclear cells (PBMCs) were isolated from active lupus patients and healthy controls at the same time point by density gradient centrifugation on Ficoll, and PBMCs were resuspended in PBS containing 1% bovine serum albumin and 0.1% sodium azide. PBMCs were resuspended in PBS containing 1% bovine serum albumin and 0.1% sodium azide. For the staining of surface antigens of cells, they were incubated with FITC-, PE-, PE-Cy7-, APC, BV711-, PE-Dazzle594-, APC-Cy7-, BV605-, or PerCP-Cy5.5-conjugated monoclonal antibodies or their isotype-control antibodies as indicated for 15 min on ice. For the staining of intracellular cytokines, they were incubated with PE-Dazzle594-, or BV421-conjugated monoclonal antibodies or their isotype-control antibodies as indicated for 30 min on ice after permeabilization and fixation.
CD1c+DCs were purified with magnetic cell sorting (MACS) using anti-CD1c (BDCA-1) micro beads according to the manufacturer's instructions. In brief, CD19+ cells were depleted using anti-CD19-coated magnetic beads, and then CD1c+ cells were isolated using biotinylated anti-CD1c and anti-biotin-beads.For proliferation assays, CD1c+DCs (1×106/well) were cultured alone or co-cultured with UC-MSCs at a ratio of 10:1, Cell Proliferation Dye eFluor™ 670 was added to the cultures. The incorporation of eFluor™ 670 in CD1c+DCs was tested by flow cytometry after 72 hr co-culture. For the detection of apoptosis, CD1c+DCs (1×10^5/well) was cultured alone or with UC-MSCs at a ratio of 10:1. Three days later, CD1c+DCs were collected, resuspended in 1×Annexin V binding buffer, and stained with FITC-annexin V and 7AAD (BD Biosciences). Annexin V+ 7AAD+ cells were detected by flow cytometry.
Fresh umbilical cords were obtained with informed consents from healthy mothers in local maternity hospitals after normal deliveries. UC-MSCs were prepared as described previously. UC-MSCs were prepared by the Stem Cell Center of Jiangsu Province (Beike Bio-Technology). The umbilical cords were rinsed in PBS with added penicillin and streptomycin, the cord blood being removed during this process. The washed cords were cut into 1-mm^2-sized pieces and floated in DMEM-LG containing 10% FBS. The pieces of cord were subsequently incubated at 37°C in humid air with 5% CO2. Nonadherent cells were removed by

washing. The medium was replaced every 3 days after the initial plating. When well-developed colonies of fibroblast-like cells appeared after 10 days, the cultures were trypsinized and passaged into a new flask for further expansion. At 80-85% confluence, the adherent cells were detached by treatment with 0.125% trypsin and 0.1% EDTA.
Cells of passages 2 to 5 were used for clinical treatment. Before infusion, cell viability was determined by trypan blue testing. The culture supernatant was analyzed for pathogenic microorganisms (bacteria and fungi) by direct cultivation analysis. Supernatant levels of alanine aminotransferase and endotoxins for each cell preparation were determined using an automatic biochemistry analyzer and by tachypleus amebocyte lysate analysis, respectively. In addition, supernatant virus indexes were determined by enzyme-linked immunosorbent assay. Cell surface labeling markers, including CD29, CD73, CD90, CD105, CD45, CD34, CD14, CD79, major histocompatibility complex class II (MHC-II) molecule and HLA-DR, were studied by flow cytometric analysis. We used good manufacturing practice conditions and clinical grade reagents to prepare the cells, and the protocol was conducted in compliance with GCP standards.

Instrument

FACS Calibur, BD-LSR Fortessa

Software

FlowJo X 10.0.7r2 software was used for analysis.

Cell population abundance

The abundance of cells for flow cytometry analysis was at least 10000 for each sample. The purity of sorted CD1c+DCs cells were typically >85% pure.

Gating strategy

Isotype control and fluorophore-positive cells were used to establish gates for each cell type. Gates were drawn to collect cells expressing either fluorophore. See the provided examples for gates used.

☒ Tick this box to confirm that a figure exemplifying the gating strategy is provided in the Supplementary Information.
